# Implementing Best Practices and Validation of Cryopreservation Techniques for Microorganisms

**DOI:** 10.1100/2012/805659

**Published:** 2012-05-02

**Authors:** David Smith, Matthew Ryan

**Affiliations:** Bioservices Team, Egham, CABI, Surrey TW20 9TY, UK

## Abstract

Authentic, well preserved living organisms are basic elements for research in the life sciences and biotechnology. They are grown and utilized in laboratories around the world and are key to many research programmes, industrial processes and training courses. They are vouchers for publications and must be available for confirmation of results, further study or reinvestigation when new technologies become available. These biological resources must be maintained without change in biological resource collections. In order to achieve best practice in the maintenance and provision of biological materials for industry, research and education the appropriate standards must be followed. Cryopreservation is often the best preservation method available to achieve these aims, allowing long term, stable storage of important microorganisms. To promulgate best practice the Organisation for Economic Development and Co-operation (OECD published the best practice guidelines for BRCs. The OECD best practice consolidated the efforts of the UK National Culture Collections, the European Common Access to Biological Resources and Information (CABRI) project consortium and the World Federation for Culture Collections. The paper discusses quality management options and reviews cryopreservation of fungi, describing how the reproducibility and quality of the technique is maintained in order to retain the full potential of fungi.

## 1. Introduction

Living organisms, their cells, or their replicable parts (e.g., genomes, plasmids, viruses, and cDNAs) are the basic elements of the life sciences and biotechnology. They are utilised in large numbers as living reference materials for testing (e.g., challenge and quality testing) and identification. Microbes are the producers of compounds, fuel and food and the tools for knowledge generation. They are grown and utilised in laboratories around the world and are key to many research programmes, industrial processes, and training courses. The biological materials on which data is generated for publication or included in databases must be available for the confirmation of results, further study, or reinvestigation when new technologies become available. These biological resources must be maintained without change to ensure reproducibility and sustainability. It is therefore the task of biological resource collections to provide these materials to their users, and, on every occasion, they must be of high quality and fulfil product claims as defined in their collection catalogues. At all times, appropriate techniques and procedures that comply with relevant national and international law must be in operation. Regular audits must be carried out to ensure that these procedures are followed and are effective. In order to achieve best practice in the maintenance and provision of biological materials for industry, research, and education, the appropriate standards must be followed. Cryopreservation is often the best preservation method available to achieve these aims, allowing long-term, stable storage of important microorganisms.

Culture collections have recognised the importance of quality management and have operated to international accepted criteria for over three decades. The World Federation for Culture Collections (WFCCs) produced guidelines which are currently in their third edition [1]. The guidance lays down criteria for the establishment and operation of culture collections, and amongst the key objectives is the use of long-term preservation techniques. The WFCCs recognise that different microorganisms often require special preservation methods in order to ensure optimal viability, storage, and purity. For security, and in order to minimise the probability of strains being lost, each strain should, whenever practical, be maintained by at least two different procedures. At least one of these should be by freeze-drying (lyophilisation) or storage at ultra-low temperature in liquid nitrogen or mechanical freezers maintaining temperatures of −140°C or lower cryopreservation; these are the best methods for minimising the risks of genetic change. In some cases, for example cell lines, where only freezing is available, duplicates should be stored in separate refrigerators with different electrical supplies (http://www.wfcc.info/guidelines/).

Single collections, national federations, and regional organisations or consortia have also developed their own more specific quality criteria but always based on the WFCC basic principles. There are also a number of quality assurance systems available that are being adopted by culture collections including those of the International Standards Organisation (ISO). These have been reviewed for suitability for application in laboratory-based collections of living cells during European projects such as European Biological Resource Centres Network (EBRCN) funded by the European Commission Framework programme 7 (QLRT-2000-00221) http://www.ebrcn.net/ and the more recent project EMbaRC-European Consortium of Microbial Resource Centres (http://www.embarc.eu/). Biological Resource centres (BRCs) must apply quality control and assurance measures to maintain the high standards necessary. These are the next generation culture collection.

The Organisation for Economic Development and Co-operation (OECD) published the best practice guidelines for BRCs (DSTI/STP/BIO(2007)9/REV1) which includes, amongst many aspects of BRC operation, controls for validation of preservation methodology and strain stability. The OECD BRC Task Force first reported in 2001, *Biological Resource Centres-Underpinning the Future of Life Sciences and Biotechnology*  (http://www.oecd.org/LongAbstract/0,3425,en_2649_34797_31685726_1_1_1_1,00.html) [2]. This report argued the need for biological resource centres, strengthened and modified to meet the requirements of the 21st century, and recommends the creation of a Global Biological Resource Centre Network (GBRCN). The OECD best practice consolidated the efforts of the UK National Culture Collections (UKNCC; http://www.ukncc.co.uk/) and the European Common Access to Biological Resources and Information (CABRI; http://www.cabri.org/) partnership via the EBRCN project. The guidance is to help ensure that biological materials are of the highest standard and authentic. The techniques used for these purposes must retain the full potential and ensure the biological material's consistency in all laboratories supplying it. These standards cover the following:

1. laboratory design and procedural requirements
2. handling, authenticity, preservation and distribution procedures
3. data recording, validation and access
4. compliance with national and international rules, regulations and policies
5. auditing and accreditation procedures.

The GBRCN demonstration project, funded by the German Federal Ministry of Research and Education (BMBF), continued the work and examined mechanisms for the adoption of the best practices. One option being discussed by candidate BRCs is the implementation of the guidance alongside ISO 9000:2000 series certification whilst the French BRCs worked with Association Française de Normalisation (AFNOR) to produce a specific French standard, NF S96-900 [3]. Whatever process is put in place, it must include optimisation of the cryopreservation procedure used to preserve the organism and testing to sufficient depth to determine if cell properties have been affected.

## 2. Quality Management in Culture Collections

The OECD BRC Task Force considered it essential that a common quality standard was developed for BRCs and that it was based upon an extension of existing guidance that had been established for microbial and cell culture collections, for example,

- the WFCC *Guidelines for the establishment and operation of collections of microorganisms* (http://www.wfcc.info/guidelines/)
- UKNCC quality management system (http://www.ukncc.co.uk/)
- common access to Biological Resources and Information (CABRI) guidelines (http://www.cabri.org/).

There are also standards that can be applied to microbiology laboratories such as good laboratory practice (GLP) and several international standards organisation norms, for example, ISO 17025, ISO Guide 25, and the ISO 9000 series. Industry is expressing the need for quality control and standards within collections. Although publications on collection management and methodology give information on protocols and procedures [4–8], the quality management system must go further and set minimum standards. Additionally, the common access to biological resources and information (CABRI) electronic catalogue project made available a set of guidelines to aid collections to put in place best practice 9. These cover critical elements in the handling, storage, characterisation and distribution of microorganisms and cell cultures, and the handling of associated information. Several collections recognised the need to implement recognised standards and have opted for the well-established ISO mechanisms (Table 1).

It soon became clear that ISO 9001:2000 was not enough to cover BRC operations; although it helped put in place good management systems, it did not address the product or the competence to produce this. Often the microorganisms that culture collections maintain, authenticate, grow, preserve, and supply are referred to as reference strains. ISO have a number of standards that apply to reference materials, and these have also been adopted and tested by culture collections. ISO 17025 *general requirements for the competence of testing and calibration laboratories* have been assessed for use by the GBRCN partners. It was shown that ISO 17025 fails to address some of the key operational requirements of a BRC. Some of these are compliance with various legal requirements in association with the handling and shipping of biological materials, the use and preparation of reagents, media, and other supplies, a strategic plan for BRC future sustainability in order to avoid the loss of biological resources, and data management and staff qualifications and competence. However, in some other aspects, it goes too far and it would demand that each process, each preservation technique, and each authentication method, the supply of strains, would have to be independently accredited. Despite this, ISO 17025 could be used by a BRC to demonstrate its competence in the preservation and supply of authentic materials.

ISO Guide 34, *general requirements for the competence of reference material producers,* has been recommended by accreditation bodies to be the most suitable for BRCs. However, this guide was written for reference material producers and used for the calibration of measuring equipment and for the evaluation or validation of measurement procedures such as pharmacopoeia standards and substances. There are some differences that may confuse or cause problems when applying it to living biological material. Assignment of property values and their uncertainties may be problematic, but it is possible that suitable values could be established for living materials. The guide also states that the reference material producer shall use documented procedures based on accepted statistical principles for the assignment of property values and lays down the procedures on which this should be based. Many of these principles cannot be applied directly to living cells, and this must be taken into account in documents that would give guidance on the accreditation procedure for BRCs. The implementation of ISO guide 34 requires the additional implementation of other guides. Amongst these is ISO Guide 31 which requires a statement of the certified property values, their meaning, and their confidence limits. It summarises the content and reason for a certificate which condenses all the information about a provided sample. Certificates are becoming increasingly necessary with the increase in the number of reference material producers and would have to be designed for each strain in the collection. Although ISO Guide 34 needs closer scrutiny, the concepts described could be applied to living reference materials.

The EU project (QLRT-2000-00221) European Biological Resource Centres Network (EBRCN) consortium recognised the need for a specific standard. The key elements of existing guidance were brought together and a European BRC Standard drafted. This was delivered to the OECD and formed the basis of the OECD best practice guidance. The latter was formulated at two levels, the general criteria that can be applied to all BRCs and secondly, at the organism domain level where specific criteria that are applied to BRCs based on the biological materials they hold were designed. The domains cover microorganisms, including viruses, animal, plant and human genetic material, cell lines, and tissues as outlined in the OECD definition. To date guidance is set for microorganisms and human-derived material but is only envisaged for BRC's holding plant and animal material.

**(i) General best practice guidelines for all BRCs covers the following**

- organisational requirements,
- equipment use, calibration, testing, and maintenance records,
- documentation management,
- data management, processing and publication,
- preparation of media and reagents,
- accession of deposits to the BRC,
- preservation and maintenance,
- supply,
- quality audit and quality review.

**(ii) Best practice guidelines for the microorganism domain covers the following:**

- staff-qualifications and training,
- hygiene and biosafety,
- equipment use, calibration, testing, and maintenance records,
- preparation of samples,
- information provided with the biological material supplied.

Additionally, specific guidance was prepared to cover potential dual use organisms and to ensure BRCs implemented practice to ensure biosecurity. Dual use is a term used in biology to refer to technology which can be used for both peaceful and harmful, for example, bioterrorism, aims.

**(iii) Best practice guidelines on biosecurity for BRCs**

- assessing biosecurity risks of biological material,
- new acquisitions/reassessment of inventory,
- biosecurity risk management practices,
- physical security of BRCs,
- security management of personnel and visitors,
- incident response plan,
- material control and accountability,
- supply and transport security.

## 3. Culture Collections and Their Transition to Biological Resource Centres

The change in how science research is conducted today utilising new technologies and information requires culture collections to adapt in order to provide the resources in a way that will facilitate their use. The adoption of international scientific and performance criteria, adding value to strain holdings and networking to share strategy, distinguishes the microbial domain Biological Resource Centre (mBRC) from the laboratory culture collection. Today culture collections must deal with the vast diversity of new genetic entities generated by life scientists as they seek to reveal the genomes of many organisms and to engineer new cells with novel genomes. Genomics leads to the amplification of biodiversity in the form of clones containing fragments of whole genomes. Sequencing the genome of a single human cell generates tens of thousands of new entities (e.g., yeast containing fragments of the human genome) that need to be conserved and distributed by BRCs. Similarly, each bacterial cell sequenced means hundreds of such new entities for BRCs. Genomics studies are generating extraordinary amounts of information and taxing the capabilities of informatics for analysing and using data. Biologists and biotechnologists will spend the next few decades understanding and exploiting the information provided by these genome-sequencing efforts. These sequence data and their byproducts, for example, genome libraries, have to be preserved and made easily accessible. The quest to obtain information on each of the thousands of genes, gene products, and other characteristics of each organism highlights the daunting task of storing, maintaining, and disseminating this information faced by BRC data banks. Similarly, many products of genetic modification, ranging from genetically engineered bacteria to transgenic plants and animals, must be preserved for scientific investigations and for commercial applications of biotechnology, as well as for regulatory and safety purposes.

These challenges require a different approach in both material and data management, and the adoption of new methodologies to ensure stability in preservation has been achieved. The culture collection faces many different challenges, and the transition to become a BRC is simply the process by which it is given tools and mechanisms to cope with them. These challenges cover the following.

- Sustainability.
- Compliance with legislation.
- Quality management.
- Information technology.
- Training and capacity building.
- Diminishing taxonomic expertise.
- Application of new technologies.
- Massive incorporation of biodiversity items.

Facing these challenges alone is not necessary; through the establishment of the global network-GBRCN, these challenges can be shared and mechanisms developed to help the BRC cope. Although it can be argued that sustainability is the prime challenge, there are many examples of how this can be achieved. Quality management has been addressed through the OECD initiative and the EMbaRC and GBRCN activities. The network itself van help access new technologies through partnerships and in the same way help access available expertise. However, it is evident that common strategy is needed to address the incorporation of biodiversity. An example of the current gap in coverage is given by the fungi. There are currently 100 000 species of fungi named, but there remains an estimated 1.4 million yet to be isolated and described. The current rate of around 1000 new species being described each year we will require another 1400 years to complete the task. This with the products of genomic studies requires us to have sound preservation technology that will cope with the variety and mass of samples. Cryopreservation has to provide the answer.

## 4. Microorganisms

Microorganisms include both prokaryotes and eukaryotes and span a wide range of organism types, they include animal, human, and plant cells in culture, algae, animal viruses, archaea, bacteria, filamentous fungi, plant viruses, plasmids, protozoa, and yeasts. They, therefore, present a challenge in their preservation. There are many considerations that must be taken into account; these include the type and structure of material to be preserved, the intended use of the material, and the facilities and expertise available. There are many preservation techniques that can be used, but three are more commonly applied to provide long-term stable storage. Freeze drying and liquid drying are often applied to bacteria and can be successful for some of the other types of microorganism, but such techniques in the main fail to work for most vegetative states [8]. Cryopreservation, therefore, plays an important role in the long-term conservation of microorganisms as adaptations to the protocol that can be made to suit each cell type.

## 5. Overview of Cryopreservation

The capacity of living organisms to survive freezing and thawing was first realised in 1663 when Henry Power successfully froze and recovered nematodes [21]. Polge et al. [24] became the first modern day scientists to report the successful freezing and viability of living organisms with avian spermatozoa. The first attempts to use cryopreservation for bacteria was in the early 1900s using liquid air [25] with cryopreservation in liquid nitrogen first noted in the 1930s [26, 27].

Cryopreservation of fungi was first noted in 1960 [28] and since then methodologies have been optimised for the vast majority of microbial groups, for example basidiomycete fungi [29–31], zygomycetes [7, 32, 33], and ascomycetes [7, 34, 35], chytrids and chromists [7].

Cryopreservation is used by almost all microbial culture collections in the developed world. Some use mechanical mechanisms to achieve low temperature, but the preferred methodology involves storing cultures in the vapour phase of liquid nitrogen. If used correctly, liquid nitrogen poses little risk. Users should ensure that safety systems are in place such as atmospheric oxygen detectors, air recirculation fans, and appropriate personal safety equipment for users. The costs of storing in liquid nitrogen can be quite high, as the liquid can be expensive although larger facilities can make economies of scale. Cryorefrigerators, safety devices, and controlled rate cooler all come at high cost. However, once stored cultures require little maintenance and can be kept safely for many years.

To reduce the risks of cryoinjury, traditional approaches for cryopreservation have involved controlled cooling at −1°C min^−1^, typically in the presence of a cryoprotectant such as glycerol, trehalose, or DMSO [8]. Cryoinjury is a result of several stresses that includes concentration effects caused by pH changes, precipitation of buffers, dissolved gases, electrolyte concentration, intracellular crystallisation resulting from loss of the water of hydration from macromolecules, and cell shrinkage [36, 37]. Membrane damage can be a result of concentration effects but may also be caused by ice damage. The physical effects of ice damage can also result in cells becoming ruptured. For microorganisms that are prone to cryoinjury or that exhibit poor viability following preservation, specific protocols can be designed to ensure optimal cryopreservation. Problem organisms include bacteria such as *Helicobacter*, fungi such as members of the Basidiomycota, and Chromists such as *Pythium* and *Saprolegnia* spp. These organisms are often termed preservation recalcitrant. The need for improved preservation procedures has given rise to the field of preservation technology. However, few collections are actively involved in preservation protocol research and development.

Some workers advocate the use of bead systems for the cryopreservation of bacteria. In this technique, bacteria are inoculated into a commercially available “bead system” which are then frozen according to the manufacturer's instructions. Beads can then be removed and directly placed onto an appropriate nutrient media. Unused beads are then often refrozen. While the method may be very simple, repeated freeze thaw cycles can potentially compromise the genetic integrity of the organisms.

### 5.1. Technology Transfer

Many technologies used for the cryopreservation of microorganisms, have been developed in the allied fields of medicine, animal, and plant cell biotechnology and zoology. The techniques employed include vitrification cryopreservation [38, 39], encapsulation [40], and cryopreservation with a host substrate [31]. Vitrification is a technique involving the application of highly concentrated, potentially toxic solutions of cryoprotectants, and has been applied to organisms of many cell types, especially plant cells [41] and human gametes [42] often using the Crytop method [43]. Typically, the vitrification solution that surrounds the cells forms an amorphous glass upon cooling; this prevents the onset of cryoinjury. Samples are rapidly cooled in liquid nitrogen which negates the need for controlled rate cooling. On resuscitation from the frozen state, samples are warmed and then “liquefy.” Care must be taken to ensure that samples do not crack which could cause physical damage to the specimen, and samples must be immediately washed to remove the toxic vitrification mixture. The technique has been applied to a range of fungi with some success [40]. Encapsulation cryopreservation is a technique where cells are embedded in calcium alginate beads prior to cryopreservation, the use of which is now well documented for microorganisms, for example, *Serpula lacrymans* [44] and monoxenically produced spores of the Glomeromycota [45]. The application of encapsulation has two main benefits; firstly the water content of cells can be reduced by osmotic treatment or drying which decreases the prospects of ice damage or concentration effects during the cooling stage of the cryopreservation procedure and secondly, it allows cells to be easily handled and manipulated by providing a suitable suspending matrix. Specimens are then rapidly “plunge” cooled with no need for controlled rate cooling.

Encapsulation and vitrification cryopreservation techniques have much potential for preserving recalcitrant microorganisms that would otherwise not be stored in the long term. However, further research and testing is required before the methods can be applied routinely to microorganisms. For obligate pathogens or mutualistic microorganisms, preservation with their growth substrate or host has been applied for many years. For example, hemp seeds have been used to support members of the Chromista when cryopreserved. This approach has been used for the microcyclic rust fungus *Puccinia spegazzini* where the teliospores were preserved on petiole tissue [31]. Similarly, seeds of the common spotted orchid (*Dactylorhiza fuchsii*) and green-winged orchid (*Anacamptis morio*) were encapsulated in alginate beads with hyphae of the basidiomycete fungus *Ceratobasidium cornigerum* with no adverse effects after cryopreservation [46]. These alternative approaches to cryopreservation have enormous potential for the large numbers of unculturable microorganisms that would otherwise not be stored by genetic resource centres.

The development of N_2_ free controlled rate coolers for use in medical cryobiology approaches is now being appraised for use in microbial cryopreservation. The Grant Asymptote EF600 is such an example; it is electrically powered by a stirling cycle cryocooler. Vials are cooled on a metallic plate which allows the cooling rate to be precisely controlled down to −80°C allowing good recovery on thawing. The cooling rates can be either linear or nonlinear, and the cycle can be suspended to allow manual seeding of ice. Initial results suggest that the application of this technology is as good as traditional methods of cryopreservation.

## 6. Implementing Best Practice and Validation of Methodology

Best practice demands that not only the modern day BRC performs authentication tests and establishes baseline information for instorage maintenance checks and validation after preservation but requires all laboratory collections to ensure validity of stored materials. The OECD best practice guidelines advise that competent persons carry out such operations and that a maintenance plan for periodic control is put in place for each organism preserved. The guidelines provide best practice at two levels, that which is applicable to all collections and BRCs holding biological material and a second level that make domain-specific recommendations, for example for the microbial resource collections. At the general level, it is recommended that biological material be preserved by at least two methods but if two distinct methods are not applicable that cryopreserved stocks be maintained in separate locations. Master cell banks must be laid down from which further stocks for distribution can be sourced. The details of the techniques are laid down in the domain-specific criteria. The guidance requires that the number of transfers or generations of original material before preservation and storage be kept to a minimum, the master stock should be created from the original material and that sufficient distribution stocks are produced to minimise the need to go back to stocks for replenishment. The material should be stored under environmental parameters that assure the stability of its properties and that detail of the inventory control, lead times, and restocking practices are documented and that duplicate collections should be maintained, preferably on another site as a “disaster” protection measure and also to avoid accidental loss. The guidance requires the validation of methods and the recording of such details. Finally quality audits and review procedures should be in place. Such practices are becoming more common in collections as automation and technology is developed to help manage such processes. In mycology, data is available on many common species to enable the selection of best protocols and assess stability, but it is far from complete.

Validation of cryopreservation methodologies between collections is not common. Individual collections often see variations in methodology to be commercially advantageous and therefore do not publicise improvements in methodologies. Some cryopreservation methodologies, particularly of fastidious microorganisms, are subject to international patent, restricting their application and thus their potential benefit for the wider scientific community. Published methods are often trialled in different laboratories, but comparisons of outputs are seldom published. However, large-scale collaborative projects between different culture collections have provided a mechanism to allow the improvement and validation of cryopreservation methodologies. For example, The EU EMbaRC (European Consortium of Microbial Resources Centres) project (sponsored by EU 7th Framework Programme, Research Infrastructures action) was established to bring together key microbial resource centres in Europe to improve, coordinate, and validate microbial resource delivery to European and International researchers from both public and private sectors. Research activity within the project was targeted at improving protocols and developing new cryotechniques for the preservation of delicate/recalcitrant strains. Work packages within the project aim to validate the methodologies developed through a regime of interlaboratory testing. This research will be complemented with a comparison of “like” strains deposited in the different partner collections. The previous EU COBRA project, *Cryopreservation and conservation of microalgae: the development of a Pan-European scientific and biotechnological resource,* involved a validation of cryopreservation regimes work package. Two cryopreservation methods, colligative cryoprotection coupled with controlled cooling and vitrification-based, encapsulation dehydration, were validated by five members of the EU Research Infrastructure consortium, COBRA, and two independent external validators http://cordis.europa.eu/search/index.cfm?fuseaction=proj.document&PJ_RCN=5251257.

## 7. Validation of Preservation Success

Postpreservation physiological or genetic changes to microorganisms following preservation have been previously recorded. To ensure that cultures have not undergone physiological or genetic change following cryopreservation, they should be examined in depth. This should be more than assessments of growth rate and culture morphology and could include analysis of metabolism or an assessment at the molecular level. A programme of tests to ensure stability of strains must be put in place. Known properties can be checked periodically, but full metabolic profile checks are seldom necessary on a regular basis. However, to be able to judge stability, a less stable property should be selected to indicate how well a strain is being maintained. Optimised techniques and standard procedures should be adhered to. It is necessary that procedures are documented, so that all coworkers and their successors can follow them and that in the future the methods and the treatments used can be traced.

There are many methods available for screening anatomical, physiological, and biochemical properties. Techniques have been derived from a wide range of different biological disciplines including biochemistry, bacteriology, mycology and ecology. The methods presented mainly provide diagnostic characters but may be extended to provide general applications such as purification, identification, and screening of properties of industrial and economic importance. Many of the techniques used to characterise fungi and bacteria can be found in Table 2. However, other methods that may be suitable include Matrix Assisted Linear Desorption Ionisation Time of Flight analysis (MALDI-TOF), and Gas Chromatography and Liquid Chromatography-Mass Spectrometry (GC-MS/LCMS) approaches.

So that comparisons of genomic or physiological integrity can be undertaken preservation, it is essential that a set of “bench mark” or “wild-type” characters should be assessed and recorded before cryopreservation. The tests must also be reproducible and established under a set of standardised conditions with respect to culture media and environmental parameters such as incubation conditions (temperature, light, humidity, etc.). Data recording is an essential prerequisite, and the recording and storage of data must be accurate and presented in a way that is interpretable by others. Where possible images, spectra, sequences, profiles, and other data formats must be stored and duplicated, preferably in a database where data should be stored together.

## 8. Summary

An overview of the impacts and consequences of implementing a quality management system on laboratory protocols and its importance has been presented. A summary of the procedures used for the validation of cryopreservation regimes within a quality management approach is presented in Table 3. Currently, only the French Standards Agency (Association Française de Normalisation) [3] has produced a standard by which to accredit BRC's of which cryopreservation procedures are a part. It is hope that other National accreditation bodies will produce similar standards in the future. Cryopreservation is the best method for maintaining the genomic integrity of microorganisms; in the future, developments and improvements in preservation methodology should allow the method to be applied for microorganisms that at the current time are difficult to maintain in BRC's.

## Figures and Tables

**Table 1:** Collections operating independent third party assessed certification or accreditation quality management systems.

Collection	System
AGO-Arocrete Group Co., Taiwan	ISO 9000:2000 series
BIOCEN (BioCC)-Centro Nacional de Biopreparados, Cuba	ISO 9000:2000 series
CABI-CAB International Genetic Resource Collection, UK	Part of services to ISO 17025
CBS-Centraalbureau voor Schimmelcultures	ISO 9000:2000 series
CCCM-Czech Culture Collection of Microorganisms	ISO 9000:2000 series
CCRC-Culture Collection and Research Center, FIRDI, Taiwan	ISO 9000:2000 series
CECT-Coleccion Espanola de Cultivos Tipo, Spain	ISO 9000:2000 series
CIP-Collection de l'Institut Pasteur, France	ISO 9000:2000 series
DSMZ-Deutsche Sammlung von Mikroorganismen und Zellkulturen, Germany	ISO 9000:2000 series
ECACC-European Collection of Cell Cultures, UK	ISO 9000:2000 series
ICLC-Interlab Cell Line Collection; Italy;	GMP
IFM-Quality Services Pty Ltd, Australia	ISO Guide 34
IHEM-Institute of Hygiene and Epidemiology, Mycology, Belgium	ISO 9000:2000 series
LMBP-Plasmid collection, Belgium	ISO 9000:2000 series
LMG-University of Gent, Belgium	ISO 9000:2000 series
MUCL-Mycology, University Louvain la Neuve, Belgium	ISO 9000:2000 series
MUM-Universidade do Minho, Uminho-MUM, Portugal	ISO 9000:2000 series
NBRC-NITE Biological Resource centre, Tsukuba, Japan	ISO 9000:2000 series
NCIMB-National Collection of Industrial, Food, Marine Bacteria, UK	ISO 9000:2000 series
NCPV-National Collection of Pathogenic Viruses, UK	ISO 9000:2000 series
NCTC-National Collection of Type Cultures, UK	ISO 9000:2000 series
NCYC-National Collection of Yeast Cultures, UK	ISO 9000:2000 series

**Table 2:** Some commonly used methods used to assess strain stability following cryopreservation.

Method	Test
Anatomical	Microscopical observation of anatomical structures. For example, spores, conidia, flagella, plastids, and hyphal form.
Culture characters	Analysis of culture morphology in plate culture. For example, pigmentation, abundance of sporulation, presence or absence of sectors, or abnormal growth
Growth rate	Measurement of radial growth of fungi and other mycelial organisms in plate culture [9]
Cell density	Cell counts at set time points using microscopical counting methods, flow cytometry of spectrophotometric approaches
Molecular integrity	PCR fingerprinting approaches (ISSR, AFLP) which assess the whole genome [10, 11]
Viability of cells	The use of chromatogenic or fluorogenic viability indicators. Many available, commonly used ones for fungi and bacteria include fluorescein diacetate (FDA), 3-(4,5-dimethylthiazol-2-yl)-2,5-diphenyltetrazolium bromide (MTT) [12] *fluorescein isothiocyanate (FITC), *FUN-1 viability staining
Enzymic capacity	APIZYM utilisation of naphthyl-bound substrates that yield a chromatogenic change [13, 14]
	4-methylumbelliferone [15–17]
Metabolic stability	High performance liquid chromatography (HPLC) of secondary metabolites [18]
	Thin layer chromatography (TLC) of secondary metabolites [18, 19]
Pathogenicity	The target organisms are inoculated onto test media with the potential control strain (or metabolite/protein extract from the control strain)/or directly onto a plant or animal, and the extent of pathogenicity and mortality are recorded.

**Table 3:** Validation of fungal cryopreservation protocols.

Criterion	Requirement	Reference
Select optimal growth conditions	To produce healthy material spores, mycelium	[20]
Measure and record baseline data for stability checks	Apply a unique identifier/strain number; select criteria to be measured: Morphological characteristics Sequence ITS region of the genome Growth rates Photomicrographs Metabolic data Genome fingerprinting techniques	[8]
Select the most appropriate preservation protocol	Optimised for organism type	[20]
Select cryoprotectant	Appropriate for the cell type	[8]
Select most appropriate cooling rate	Thermometer calibrated to a standard	[8]
Select most appropriate storage temperature	Temperature below–140°C, monitored and recorded	[21, 22]
Select most appropriate thawing protocol	A rate appropriate to cell type in calibrated and controlled equipment	[8]
Prepare master and distribution stocks	High recovery No contamination Authentic: morphology; phenotypic and molecular integrity	[23]

Method validation		
Performing blind tests	Central laboratory sends unknown organism to collections with limited data and results after above the process compared	Example of such a system is in the public health laboratories for diagnostics
Reproducibility check	Comparing results of the same method at different times Comparison of results obtained with different methods Comparison of results obtained with different operators	[23]
Equipment calibration	All equipment must be regularly serviced and gauges and meters calibrated to recognised standards	[23]
Recording parameters	Daily records of temperature readings of incubators and cryostorage units to ensure that they remain within set parameters	ISO standards
